# An interdisciplinary and participatory methodology to improve user acceptability of root, tuber and banana varieties

**DOI:** 10.1111/ijfs.14680

**Published:** 2020-09-04

**Authors:** Lora Forsythe, Hale Tufan, Alexandre Bouniol, Ulrich Kleih, Geneviève Fliedel

**Affiliations:** ^1^ Natural Resources Institute University of Greenwich Central Avenue, Chatham Maritime Kent ME4 4TB UK; ^2^ College of Agriculture and Life Sciences Cornell University 215 Garden Avenue Ithaca NY 14853 USA; ^3^ CIRAD UMR QUALISUD Cotonou 01 BP 526 Benin; ^4^ Qualisud, Univ Montpellier CIRAD Montpellier SupAgro Univ d'Avignon, Univ de La Réunion Montpellier 34398 France; ^5^ Faculté des Sciences Agronomiques Laboratoire de Sciences des Aliments Université d’Abomey‐Calavi Jéricho 03 BP 2819 Benin; ^6^ CIRAD UMR QUALISUD F‐34398 Montpellier France

**Keywords:** Breeding, consumer acceptance, Food Product Profile, processing, quality characteristics

## Abstract

Breeding programmes for root, tuber and banana (RTB) crops have traditionally considered consumer demand for quality characteristics as low priority against other considerations such as yield and disease resistance. This has contributed to low levels of adoption of new varieties and its potential benefits. To address these challenges, an interdisciplinary five‐step methodology was developed to identify demand for quality characteristics among diverse user groups along the food chain. The methodology includes an evidence review, consultations with key informants and rural communities, processing diagnosis with experienced processors and consumer testing in urban and rural areas. Quality characteristics are then prioritised into a Food Product Profile by user group to inform further work of biochemists and breeders in developing improved selection tools. This initiative presents a new basis to understand consumer preferences for RTB crops. The methodology is currently being applied in projects in sub‐Saharan Africa and is applicable globally.

## Graphical abstract

To address issues of low adoption of new root, tuber and banana (RTB) varieties, consumer demand for quality characteristics should be considered and integrated into crop breeding programmes. This paper presents an interdisciplinary five‐step methodology to identify demand for quality characteristics among diverse user groups along the food chain.

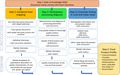

## Introduction

In the past few decades, there have been significant improvements to food security in sub‐Saharan Africa (SSA). Root, tuber and banana (RTB)^1^ breeding programmes have been an important contributor to this improvement, in part by developing new high‐yielding and disease‐resistant varieties (Evenson & Gollin, 2003). However, processor and consumer demand for quality characteristics of RTB food products have received lower priority in breeding programmes, which has impacted negatively on the adoption of new varieties throughout the continent (see Thiele *et al*., this issue). To address this *adoption gap*, there is increasing attention of researchers to support breeding programmes to become more demand‐led and take on a full food chain perspective.

RTB crops and products are vitally important for household food security and a significant component of incomes throughout SSA (Petsakos et al., 2019). There are dozens of products derived from RTB crops consumed daily. Some products of particular importance in West Africa are cassava‐based products, gari, fufu and attièkè, and in East Africa, products such as matooke, boiled cassava and sweet potato are particularly important. These products are mainly consumed at home or sold at local markets, with regional or even local preferences (Orr *et al*., 2018; Teeken *et al*., 2018). The demand for quality characteristics associated with these products is equally diverse and strongly influenced by the manner of consumption and regional preferences. Variety, agro‐ecological conditions, crop and product management, and processing steps, among other factors, impact on quality (see Thiele *et al*., this issue; Orr *et al*., 2018; Teeken *et al*., 2018). Different *users* of the crop and product (e.g. producers, processors, retailers and direct consumers) often have several specific characteristics that they prefer, depending on their role in the food chain (Efisue *et al*., 2008; Orr *et al*., 2018). For example, a producer may prioritise a high‐yielding sweet potato variety that produces large roots, and a processor may prefer a variety with little fibre and that is sweet in taste (Mudege & Grant, 2017). Some characteristics may be *non‐negotiable* for a user – meaning that the user only accepts a variety if it contains a specific quality characteristic. For example, a cassava variety that is high in cyanogenic potential would not be adopted in the market segment for fresh boiled roots, even if superior in other characteristics.

Gender and social context also play important roles in influencing the demand for certain quality characteristics. Men and women, even in the same household, have different interests in how the crop is used, what products are made and what markets it is sold to (Chambers & Momsen, 2007; Forsythe *et al*., 2015; Forsythe *et al*., 2016). Gender analysis of the preferences for quality characteristics shows that by and large, preferences follow gender divisions of labour. Women more often mentioned food security, production and use‐related characteristics, while men mentioned fewer characteristics focused on production and marketing (Weltzien, *et al*., 2020). RTB crops follow similar patterns, including cassava (Teeken *et al*, 2018), banana (Marimo et al., 2019) and sweet potato (Mudege & Grant, 2017).

While the diversity in demand for RTB product quality characteristics is a significant challenge, this methodology aims to establish a process of consultation with user groups that creates a Food Product Profile – prioritised quality characteristics for an RTB food product that reflects demand for diverse sets of users along the food chain. The profile will then inform biochemists about important quality characteristics, preferred or nonpreferred among a range of users, for later translation into physical or chemical components, followed by effective trait selection within new varieties by breeders. Subsequent papers in the Special Issue present the findings from use of the methodology for a range of RTB products.

The foundation of the approach is based on Fliedel et al. (2016) who developed a new approach for better assessing the adoption of new cassava genotypes, in view of providing information to breeders early in varietal improvement programmes. It involved several successive steps, such as qualitative surveys all along the food chain to identify quality criteria of a good cassava crop and product, effective participation of processors to identify the ability of new genotypes to make a good product and a ‘all‐in‐one’ method coupling hedonic test, JAR ‘just‐all‐right’ test and CATA ‘check‐all‐that‐apply’ table to assess the acceptability and preferences of products by a large number of consumers.

The methodology outlined here expands the Fliedel et al. (2016) approach in five progressive steps woven together in a set of participatory tools, applied along the food chain for the product. The methodology is novel in its strong interdisciplinary approach: food science, gender, economics and plant breeding were key disciplines involved in developing the methods. The methodology was adapted based on the significant and iterative input from interdisciplinary teams of our partner organisations as part of the RTBfoods project. The tools were further adapted by teams to reflect the context, product, their experience and expertise. The tools and methods are expected to evolve further with lessons learnt from the partners. Research findings from some of the teams are presented as part of this special issue.

The main research question addressed in the methodology was: *What are the quality characteristics driven by users’ demand and how can these be used to construct a Food Product Profile?* This question was broken down into the following sub‐questions:

- Who are the different users and markets in the crop and product food chains, and what are the preferred quality characteristics associated with the users?
- What are the different quality characteristics of the crop after harvesting, of the crop during processing and of the final product? What are the key processing steps to make a high‐quality product? What are the characteristics of a high‐quality product?
- What are the gender dimensions of the crop and product food chains and preferences of different user groups? At the household level, are there trade‐offs among product uses and quality characteristics, and for whom?
- What is the prioritisation of quality characteristics by gender, region and other possible social–economic segments?

This paper provides an overview of the methodology using extensive reference to the manuals developed for the RTBfoods project (https://rtbfoods.cirad.fr). The manuals describe the methodology in detail, and the terminology and methods have been adapted for broader application in this paper. Links to the manuals are provided in the paper and References section.

## Methods

The methodology for developing a Food Product Profile follows five progressive steps:

- Step 1. Research teams conducted a state of knowledge (SOK) review to establish what is known about the product and the gaps in knowledge in relation to food science, gender and markets in the country context, and to establish the scope of the further studies.
- Step 2. Experts carried out a gendered food mapping exercise in communities to identify the different uses of the crop by different users (e.g. producers, processors, consumers and local retailers) and the associated quality characteristics. The study also investigated gender and market dynamics in relation to the crop and product, and their quality characteristics. At this stage, the first draft of the Food Product Profile containing prioritised quality characteristics by user group is produced, taking into account gender and livelihood context.
- Step 3. Teams conducted a participatory processing diagnosis with experienced processors. Both preferred and nonpreferred varieties were included to provide a wide range of technological and physico‐chemical characteristics. Processors provided feedback on the varieties before processing, during each processing step and after processing to identify quality characteristics of the crop and product. Processing parameters were measured at each step. New quality characteristics from this step are added to the Food Product Profile.
- Step 4. Consumer testing was conducted with approximately 300 consumers in rural and urban areas, to provide a better understanding of consumer demand and to obtain a sensory mapping of the overall liking of each product that could be related to most liked and least liked characteristics used by each consumer to describe the product. At this stage, new quality characteristics and their prioritisation are added to the Food Product Profile.
- Step 5. The Food Product Profile is then finalised with the interdisciplinary team and transferred to biochemists and breeders for feedback and ultimately to develop improved selection criteria and methods.

**Figure 1:**
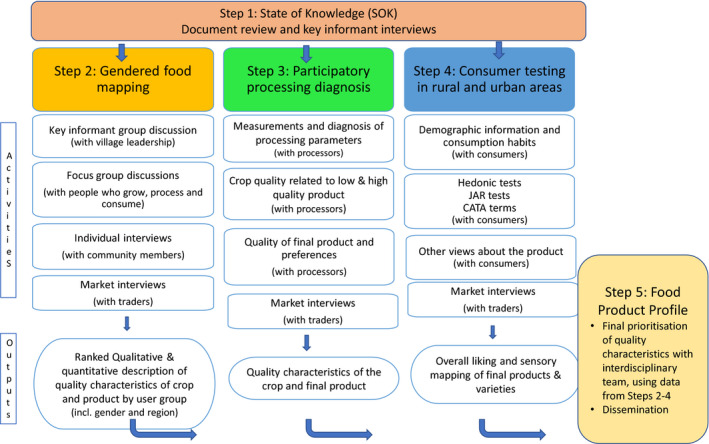
Overview of 5‐step methodology for Food Product Profile development.

Fig. 1 provides an overview of the five‐step methodology.

Research teams obtained ethical approval prior to the fieldwork. The research respected the rules of informed and written consent, voluntary participation and anonymity. Food samples were prepared according to good hygiene and manufacturing practices.

The remainder of this section provides additional details on each step, with links to the manuals, which provide full details.

### Step 1: State of knowledge (SOK)

The research process started with a SOK exercise with the following objectives: i) to establish what is known about the product, both documented and undocumented; ii) to identify gaps in knowledge; and iii) to determine scope of the studies that follow (e.g. important geographic areas with high consumption, characteristics of the food chain for sampling). Methods for the SOK included a literature review of peer‐reviewed and grey literature, and key informant interviews with experts of the RTB crop and/or product (e.g. leaders of marketing association, consumer board and Ministry of Agriculture) to obtain unpublished insights.

The SOK was divided into three disciplinary modules with the following aims:

1. The *food science module* aims to establish the important quality characteristics of a product, from raw material to final product, and processing method(s).
2. The *gender and social context module* aims to collect information on the broader social context and gender dynamics of the regions and identify user groups along the product’s food chain, drawing on the Gender Dimensions Framework described by Rubin *et al*. (2009) and Rubin & Manfre (2014).
3. The *demand module* seeks to identify demand segments for the product, defining for whom and where the demand for the product is located, and the quality characteristics associated with the product, drawing on a Segmenting‐Targeting‐Positioning (STP) framework (Orr *et al*., 2018).

The results of the SOK then informed the sampling frame for further studies and adaptation of fieldwork tools to respond to local context, current knowledge and gaps in evidence.

### Step 2: Gendered food mapping

Gendered food mapping involved a gender and market analysis along the product’s food chain in rural areas, including consultation with people who grow, process and consume the crop and product (Moser, 1989, 1993; Rubin *et al*., 2009; Rubin & Manfre, 2014; Orr *et al*., 2018). The aims of the study were to:

- Understand who is producing, processing, selling and consuming the crop and product, from a gendered perspective
- Understand the multiple uses and products of the crop and possible trade‐offs among uses
- Identify the most and least liked quality characteristics for the crop and product and how they are prioritised by different user groups
- Understand how gender influences preferences and prioritisation for quality characteristics

Importantly, the exercise collected data on all uses and quality characteristics regarding the *crop*, not just a derived food product, to identify any trade‐offs in the uses or quality characteristics preferred by user groups. For example, in southwest Nigeria, gender differences in cassava markets require different quality characteristics: men prefer varieties with high starch content to sell to industry, while women process and sell *gari* (a granular coarse flour), where other qualities may be more important, such as ease of peeling (Forsythe *et al*., 2015, 2016).

Gendered food mapping should be conducted in a minimum of two regions, selected according to their importance for production, processing and/or marketing of the product and its variations. Four rural communities in each region (total of eight) were selected randomly for the study. In each community, multiple methods were used. Key informant group discussions (one in each community) were held with community leaders to provide an overview of the community, livelihoods and the role of the crop and product in the household economy. Following this, focus group discussions (at least two in each community) were held separately with men and women who grow, and possibly process the crop, to understand the different uses and the demand for quality characteristics associated with the crop and its products.

Eighty interviews with randomly selected processors were conducted, as a minimum sample size to conduct quantitative analysis. Processors individually ranked quality characteristics in importance (using simple or pairwise ranking), for the raw material, at each stage of processing and for the final product. Data on the least liked quality characteristics; technological, physico‐chemical and sensory characteristics; gender roles; household decision‐making; and marketing information were also collected.

Economists conducted market interviews with purposively selected traders and retailers to collect data on different consumer groups and their preferences for particular quality characteristics and varieties, and projected future trends of the product. The following market interviews were conducted: at least eight in rural areas, 10‐15 in towns and 30 with retailers in towns and urban areas.

At this stage, the first draft of the Food Product Profile is produced: separate tables of prioritised quality characteristics, one each for different user groups (for men and women, for each region and according to any other factors important in the context) (refer to Table 1).

**Table 1:** The food product profile

	A	B	C	D	E	F	G	H
	Type of characteristic by sensory category Indicate if the table is for a specific user group: (e.g. men, women, Region 1, Region 2)	High‐quality characteristics Characteristics that give a good, high‐quality product	Indicator of high‐quality characteristic how respondents assess (evaluate, feel) the characteristic	Priority of high‐quality characteristic Indicate the rank, and note if simple or pairwise ranking	Low‐quality characteristics Characteristics that give a poor quality product	Indicator of Low‐quality characteristic how respondents assess (evaluate, feel) the characteristic	Varieties – GOOD Scientific names and indicate (L) local, (O) older released variety or (N) new variety released	Varieties – INFERIOR Variety names and indicate if local, released variety (and year it was released) and experimental variety to increase variability
1	***Raw material characteristics*** *for product quality (agronomic, postharvest)*							
2	***Processing characteristics*** *of raw material for the product quality during processing (technological, physico‐chemical)*							
3	***Characteristics of raw final product***							
	*To look at*							
	*To touch*							
	*To smell*							
	*To taste*							
	*Texture in mouth*							
4	***Characteristics of cooked/ready‐to‐eat final product*** `(sensory)							
	*To look at*							
	*To touch*							
	*To smell*							
	*To taste*							
	*Texture in mouth*							

### Step 3: Participatory processing diagnosis and quality characteristics

A participatory processing diagnosis was carried out with a group of processors, in processing centres or communities, to assess the processing ability of four to five crop varieties that are very different in technological and sensory characteristics. The objective of this step was to collect all the quality characteristics referred to by the processors, while they are processing the *most liked* and *least liked* varieties. The processors were interviewed and provided their opinion on the varieties before and during processing, and on the final products after processing. Variability in quality of the varieties was necessary to understand processors’ demand and enable the processors to express their needs, to identify the reasons they may adopt or reject a variety, and to describe with precision the crop characteristics that result in a good and bad product, and the characteristics of these good and bad products. Variability can be obtained by processing local varieties known for their ability (or unsuitability) to give a high (or a poor)‐quality product, but also by processing new genotypes, unknown by the processors, and with very different characteristics compared to local varieties.

As described by Bouniol *et al*. (2017), this step aimed also to: (i) give a general processing flowsheet of the crop and identify the key processing unit operations important in the quality of final products; (ii) measure technological parameters such as yield, peeling time and cooking time at each step of the process to assess the technological properties of each variety compared to local ones; and (iii) produce final products with different quality characteristics that will be used in the third step for consumer testing.

The processing demonstrations were conducted in processing centres, at least one in each region. For some products, such as gari, processing centres are located in small towns. Other products, such as boiled products, are prepared at the household level, and thus, the demonstrations were conducted in communities.

Importantly, this step generates new quality characteristics often not mentioned in Step 2. This is possible if the varieties chosen for the participatory diagnosis are very different in quality and that some varieties are unknown to the processors. This purposive introduction of wide variability to the processors should generate very good, possibly intermediate, and bad quality products, thereby eliciting a range of opinions on the quality characteristics of varieties and products. These good and bad quality characteristics are relevant for biochemists, even if they are cited by a small sample of processors who are involved in the processing diagnosis. Biochemists will analyse the varieties to translate these good and bad quality characteristics into simple physico‐chemical components, which are used by breeders to inform their breeding methods and selection criteria.

The new quality characteristics of the crop and product collected during this step are added to the draft Food Product Profile (Step 5). Results from the processing diagnosis were then used to develop the questionnaire for Step 4, Consumer Testing.

### Step 4: Consumer studies in rural and urban areas

This step aimed to understand consumer demand through an *all‐in‐one* method that included hedonic tests, a CATA question and *j*JAR tests (Fliedel *et al*., 2014; Monteiro *et al*., 2017). A large number of consumers were invited to taste the four to five products made in Step 3, from varieties with very different quality characteristics.

As preferences may vary by the type of consumer group, it is recommended that the sample include rural and urban areas (150 interviews in a primary centre/city and another 150 interviews among four rural communities previously visited in Step 2, gendered food mapping), an equal number of women and men, and to sample different locations of the city to increase representation of various socio‐economic and ethnic groups.

Consumers (approximately 300) were asked to taste each product individually, one after the other and in a random order, and score the overall liking using a nine‐point hedonic scale (1 = *extremely dislike*; 9 = *extremely like*). They were also asked about their perceptions of the intensity of two to four characteristics of the products identified as important in the previous steps, using the 3‐point JAR scale (1 = too *weak (TW)*; 2 = *just about right* (JAR); and 3 = *too strong (TS))*. Then, consumers were asked to select quality characteristics in a CATA table (Ares & Jaeger, 2013) that describes each product by the most liked and least liked sensory characteristics collected in the previous steps. A choice of 20‐25 characteristics is recommended and should refer to the appearance, odour, texture between fingers, taste, texture in mouth and aftertaste of the final products. Finally, consumers were invited to give their opinions and preferences on the products. The four to five products must have a wide range of expression of sensory characteristics so that their differences can be detected by consumers. The combined results show the most and least liked sensory characteristics for consumers.

This step identified the relationships between hedonic overall liking scores for each product and the frequencies of citation of each CATA sensory characteristic by consumers. It provided a clear mapping of the most liked products and their associated high‐quality characteristics, the least liked products and their associated lower quality characteristics, and in the middle intermediate‐quality products. Biochemists will use the sensory map to analyse all the physico‐chemical compounds of the four to five products, and the corresponding varieties, to translate the most and least liked characteristics into simple physico‐chemical components for breeders.

This mapping will be added to the draft Food Product Profile from Step 4.

### Step 5: Finalisation of the Food Product Profile

The final step produced a completed Food Product Profile, namely the prioritised quality characteristics using evidence from Steps 2 to 4. The prioritisation is important as it indicates the *must‐have* characteristics – it may not be possible to have a variety with all the desired *good* characteristics and none of the *inferior* ones. The process for final prioritisation of quality characteristics is based on number of citations and/or the weighted aggregation of rankings mentioned in the different steps of an assessment. This is then assessed by the interdisciplinary fieldwork team according to: i) visioning – exploration of what type of variety they would want to deliver and its possible impact and ii) identification of important preferences or non‐negotiables for selected groups, particularly for women. Possible negative impacts associated with quality traits also must be taken into account. Teams were asked to document their decisions citing evidence from their research (qualitative or quantitative) and other sources.

Other important features of the profile are *high‐* and *low‐quality* characteristics and their indicators, and *good* and *inferior* varieties associated with each characteristic. The quality characteristics were listed according to those associated with the raw material, processing of the raw material, the raw final product and the cooked/ready‐to‐eat final product. Refer to Table 1 below. At this stage, the Food Product Profile is considered to be ready for use by biochemists and breeders. The process is iterative. Both the methodology and the results benefit from continual improvement and updating as the interdisciplinary team develops or encounters new information.

### Data analysis

Since there are extensive qualitative and quantitative data gathered in Steps 2, 3 and 4 of the methodology, data analysis is complex. Its presentation in meaningful form is essential to achieve Food Product Profiles that can be effectively used by biochemists and breeders. Each manual includes recommendations on how to summarise and analyse data for most effective use.

For Step 2, *Gender food mapping*, the focus group discussions and interviews were transcribed, and qualitative and quantitative data were inputed into an Excel database. Excel was used as it was the most accessible software for the research teams, but qualitative software such as Atlas.ti and NVivo are recommended. Qualitative data were coded according to the interview guide, and relationships between concepts and categories (e.g. quality characteristics and their detailed description, trade‐offs between different varieties used and household decision‐making) were identified through comparison until no new findings could be derived from the analysis. Basic quantitative techniques were conducted, with some research teams undertaking more advanced techniques. To complete the Step 2 Food Product Profile Table, quality characteristics and varieties are inputted into the table based on their citation. High‐quality characteristics are ranked in order of importance based on the aggregated ranking from individual interviews. Separate Tables are produced for men, women, by region, and other important factors according to the context, to identify different preferences in characteristics and their prioritisation. However, it is important to note that as the people who process the product are disproportionately represented by women, who made up the large part of the sample, sex‐based comparison was not possible for all products.

For Step 3, *Processing diagnosis*, qualitative and quantitative data were collected. Qualitative data were collected during processor interviews using a questionnaire on quality characteristics and processing: *Discussion guideline with processors before, during and after processing*. Quantitative data were collected by measuring several parameters to compare the processing ability of each variety and assess its technological properties. Typically, but also depending on the crop/product process with different unit operations, the parameters to be measured can include the dry matter losses, the duration of each unit operation, the evolution of pH and temperature during fermentation, the evolution of the cooking temperature and the evolution of the yield during the whole process.

For Step 4, *Consumer studies*, an analysis of variance (ANOVA) is carried out to identify whether significant differences of overall liking scores are observed between the four to five products tasted by consumers. An effect such as region or gender can be studied. Multiple pairwise comparisons are undertaken using the Tukey test with a confidence interval of 95% at p < 0.05 (n = 300 consumers). For each product, the number of consumers who have found each characteristic just about right (JAR), or too weak (TW) or too strong (TS) is counted, and the percentage of consumers (n = 300) who have scored these specific characteristics is determined. A multifactorial analysis is used to show the relationships between frequencies of citation of CATA sensory characteristics and the mean overall liking scores for each product. All statistical analyses can be performed using XLSTAT 2019 (Addinsoft).

### Lessons learned

#### An integrated field approach

The progressive nature of the steps in the methodology constitutes an integrated field approach. Step 2 benefits from the results of Step 1, as the latter provides the scope for the study and the gaps in research. Step 2 provides a set of ranked quality characteristics from users who play different roles in the food chain, and in‐depth context of the research. Step 3 provides an opportunity to identify more quality characteristics in‐depth with experienced processors, who play an important interface position: a close link with agricultural production (knowledge of the characteristics of raw materials) and with the market and consumers (knowledge of the qualities expected by the consumer). The development of questionnaires and the implementation of Step 4 thus benefit from the results and observations of Step 3, and Step 4 provides robust data on preferences regarding the final product among a diverse set of consumers. Data from the different steps are then triangulated to obtain statistically sound results for the Food Product Profile. The integrated methodology enables a deep understanding of the quality characteristics, translating tacit knowledge into data that can be further investigated by scientists (Polanyi, 1966).

#### Sampling by role in value chain or by gender

The methodology includes the collection of sex‐disaggregated quantitative data and qualitative data for gender analysis. This is to compare differences in preferences for quality characteristics, their prioritisation, potential *trade‐offs* and their linkage to gender roles and agency. The individual interviews in Step 2 provide these data points and require that the individual be knowledgeable about the product and its processing. However, due to gender roles that strongly associate women with processing, this approach has the *de facto* result of a sample including mainly women, and therefore, quality characteristic preferences cannot be sex‐disaggregated. Researchers will need to clearly establish at the onset what gender‐related questions they will ask and attempt to answer, and with what type of data.

#### Good practice for qualitative research methods and interdisciplinary teams

Documenting RTB crop postharvest and consumption characteristics requires open‐ended inquiry and capturing verbatim quotes, using exact words as the community expresses data needs, in order to go beyond broad descriptions such as *sour* or *easy to peel* for important characteristics. For example, surveys should add value by asking for detail on the *type of sour*, and indicators of *sourness* or *peelability*. It is essential to have interviewers who are fluent in local languages, in addition to intimate familiarity with the methodology to ensure high‐quality data. Research teams should be interdisciplinary. Including food scientists, plant breeders and social scientists is important to raise considerations from their disciplinary perspectives.

## Conclusions

The interdisciplinary and participatory methodology is unique in its design of integrated research activities and delivery of results. In addition, the collaborative nature of the approach creates the space for research teams to adapt and use the tools in diverse and evolving contexts. The five‐step, integrated method provides an opportunity for rich data collection on the demand for RTB crop and product quality characteristics tethered to socio‐economic information in a robust manner. The results will support breeding programmes in their efforts to respond to the diverse and multifaceted needs of consumers and others in the food chain.

## Author contribution

**Lora Forsythe:** Conceptualization (equal); Methodology (equal). **Hale Tufan:** Conceptualization (lead); Methodology (equal). **Ulrich Kleih:** Conceptualization (equal); Methodology (equal). **Alexandre Bouniol:** Conceptualization (equal); Methodology (equal). **Genevieve Fliedel:** Conceptualization (lead); Methodology (lead).

## Conflict of interest

The authors declare no conflicts of interests.

## Ethical approval

Research teams obtained ethical approval prior to the fieldwork from their respective institutions. The research teams respected the rules of informed and written consent, voluntary participation and anonymity. Food samples were prepared by research teams according to good hygiene and manufacturing practices.

### Peer Review

The peer review history for this article is available at https://publons.com/publon/10.1111/ijfs.14680.

## Data Availability

The methodology presented in this paper is openly available in Agritrop open repository of CIRAD publications. The manuals can be accessed through the DOIs listed below. Forsythe, L., Fliedel, G., Tufan, H. (2018). RTBfoods Step 1: State of knowledge guidance document. Montpellier, France: CIRAD‐RTBfoods Project. https://doi.org/10.18167/agritrop/00568. Forsythe, L., Fliedel, G., Tufan, H., Kleih, U. (2018). RTBfoods Step 2: Gendered food mapping. Montpellier, France: CIRAD‐RTBfoods Project. https://doi.org/10.18167/agritrop/00569. Fliedel, G., Tufan, H., Bouniol. A., Forsythe, L. (2018). RTBfoods Step 3: Participatory processing diagnosis and quality characteristics. Montpellier, France: CIRAD‐RTBfoods Project. https://doi.org/10.18167/agritrop/00570. Fliedel, G., Kleih, U., Bechoff, A., Forsythe, L. (2018). RTBfoods Step 4: Consumer testing in rural and urban areas. Montpellier, France: CIRAD‐RTBfoods Project. https://doi.org/10.18167/agritrop/00571

## Note

^1^ Main RTB crops referenced in this paper are cassava, potato, sweet potato, yam, starchy (or cooking) banana and sweet (or dessert) banana.
